# Progression of Pro23His Retinopathy in a Miniature Swine Model of Retinitis Pigmentosa

**DOI:** 10.1167/tvst.6.2.4

**Published:** 2017-03-15

**Authors:** Patrick A. Scott, Juan P. Fernandez de Castro, Paul J. DeMarco, Jason W. Ross, Josephat Njoka, Eric Walters, Randall S. Prather, Maureen A. McCall, Henry J. Kaplan

**Affiliations:** 1 Department of Ophthalmology & Visual Sciences, University of Louisville, Louisville, KY, USA; 2 Department of Anatomical Sciences and Neurobiology, University of Louisville, Louisville, KY, USA; 3 Department of Psychological and Brain Sciences, University of Louisville, Louisville, KY, USA; 4 Department of Animal Science, Iowa State University, Ames, IA, USA; 5 Division of Animal Science, University of Missouri-Columbia, Columbia, MO, USA; 6 Department of National Swine Resource and Research Center, University of Missouri-Columbia, Columbia, MO, USA; 7 Department of Microbiology and Immunology, University of Louisville, Louisville, KY, USA

**Keywords:** retinitis pigmentosa, retinal degeneration, swine

## Abstract

**Purpose:**

We characterize the progression of retinopathy in Filial 1 (F1) progeny of a transgenic (Tg) founder miniswine exhibiting severe Pro23His (P23H) retinopathy.

**Methods:**

The F1 TgP23H miniswine progeny were created by crossing TgP23H founder miniswine 53-1 with wild type (WT) inbred miniature swine. Scotopic (rod-driven) and photopic (cone-driven) retinal functions were evaluated in F1 TgP23H and WT littermates using full field electroretinograms (ffERGs) at 1, 2, 3, 6, 9, 12, and 18 months of age, as well as the Tg founder miniswine at 6 years of age. Miniswine were euthanized and their retinas processed for morphologic evaluation at the light and electron microscopic level. Retinal morphology of a 36-month-old Tg miniswine also was examined.

**Results:**

Wild type littermates reached mature scotopic and photopic retinal function by 3 months, while TgP23H miniswine showed abnormal scotopic ffERGs at the earliest time point, 1 month, and depressed photopic ffERGs after 2 months. Rod and cone photoreceptors (PR) exhibited morphologic abnormalities and dropout from the outer nuclear layer at 1 month, with only a monolayer of cone PR somata remaining after 2 months. The retinas showed progressive neural remodeling of the outer retina that included dendritic retraction of rod bipolar cells and glial seal formation by Müller cells. The TgP23H founder miniswine showed cone PR with relatively intact morphology exclusive to the area centralis.

**Conclusions:**

The F1 Tg miniswine and the TgP23H founder miniswine exhibit similar retinopathy.

**Translational Relevance:**

TgP23H miniswine are a useful large-eye model to study pathogenesis and preservation cone PRs in humans with retinitis pigmentosa.

## Introduction

Retinitis pigmentosa (RP) is a collective term for a group of heterogeneous retinal degenerations that cause irreversible blindness and affect 1 in 3500 people worldwide.^1^ The proline-23-histidine (P23H) rhodopsin (*RHO*) gene mutation underlies the most common form of RP in North America and accounts for approximately 8.5% of all RP cases.^2^

Multiple transgenic (Tg) models of P23H retinopathy have been made in rodents,^3–5^ with the recently described P23H rhodopsin knock-in mouse^6^ having a clinical phenotype very similar to that of man. Despite the insights that rodent models have provided their eyes do not have an area of retinal specialization populated extensively by cone photoreceptors. Lack of a cone-rich area of retinal specialization is a limitation of murine models for studying the pathogenesis and treatment of cone photoreceptor degeneration in the later stages of RP. In contrast, swine have a cone-rich area of retinal specialization, the area centralis, which resembles the human macula.^7–9^ Swine models of RP^10–11^ exhibit functional and morphologic characteristics^12–14^ similar to the human phenotype. The only major drawback of swine models for general use in laboratory work and long-term projects is their large size. To circumvent this limitation, we developed Tg founder miniature swine that express the P23H *RHO* gene mutation.^11^

In our previous study,^11^ full-field electroretinography (ffERG) was used to track changes in scotopic and photopic vision out to 24 months in six TgP23H founders, while retinal histology was examined only at time of death (12–22 months). TgP23H founders showed variation in disease progression and severity and were categorized as either moderate or severely affected based on ffERG assessments. Given the variability in the progression and severity of retinopathy in TgP23H founders, characterizing the functional and morphologic changes that occur to the retina during P23H retinopathy will allow us to determine whether Filial 1 (F1) Tg offspring consistently recapitulate retinopathy observed in Tg founder miniswine. A large-eye model of RP that shows consistency in progression and severity from generation-to-generation and between Tg littermates will be very useful for development and implementation of therapeutic strategies that prevent photopic vision loss. Therapeutic strategies (e.g., cell based or gene therapy) that delay/inhibit/rescue retinal degeneration do not share the same window of opportunity and their effectiveness depends upon the stage of retinopathy. That said, our immediate objective in this project was to characterize progression of retinopathy in F1offspring of our most severely affected TgP23H miniswine founder 53-1. As a corollary to our main study, we assessed retinal function in TgP23H miniswine founder 53-1 (6 years of age), followed by examination of retinal morphology. We showed that Tg F1 progeny recapitulate functional and morphologic features similar to TgP23H miniswine founder 53-1, which exhibits an RP phenotype similar to the human form of the disease.

## Materials and Methods

### Swine

Generation, genotyping, and clinical examination of TgP23H miniswine has been described previously.^11^ A total of 44 animals hwere included in this study. F1 progeny of TgP23H miniswine founder 53-1 (RRID:NSRRC:0017) were produced by crossing with inbred WT miniswine. Three WT and three age-matched Tg littermates were tested with ffERGs and immediately euthanized at 1, 2, 3, 6, 9, 12, and 18 months, and their eyes enucleated and processed for morphologic studies. Eyes from a 36-month-old Tg miniswine and miniswine founder 53-1 also were included in this study. Eyes from the 36 month-old Tg miniswine were fixed in 2.5% formaldehyde/2.5% glutaraldehyde mixture and because of this morphologic examination of the retina was limited to light and transmission electron microscopy. All experimental protocols were approved by the University of Louisville Institutional Animal Care and Use Committee and adhere to the ARVO Statement for Use of Animals in Ophthalmic and Vision Research.

### Retinal Electroretinography (ERG)

Sedation and retinal fundus examination of animals has been described previously.^13^ The ffERG was recorded using a UTAS ERG system with a BigShot Ganzfeld (LKC Technologies, Inc., Gaithersburg, MD) stimulator. The swine's head was placed inside of the Ganzfeld bowl and the ERG measured bilaterally using monopolar contact lens electrodes (ERG-Jet electrodes; Fabrinal SA, La Chaux-de-Fonds, Switzerland) placed on the cornea. A disposable monopolar EMG needle electrode, GRD-SAF 12 mm × 29 gauge (The Electrode Store, Buckley, WA) was placed behind the ear and the reference needle electrode was on the midline of the forehead. After 20 minutes of dark-adaptation the ffERG was recorded to strobe flash intensities of −24 dB with an interstimulus interval (ISI) of 2 seconds (15 samples) to isolate the rod scotopic response. The animal then was light adapted for 10 minutes with a 20 cd/m2 background and responses recorded at 0 dB and 0.5 seconds ISI (30 samples) to measure the standard photopic cone response. Finally, the response to a 0 dB 30 Hz flicker flash (30 samples) was measured. Individual samples were analyzed to find aberrant waveforms that are rejected and then averaged into a single waveform to measure the a-wave (baseline to trough) and b-wave (peak to trough) amplitudes and latencies.

As in our previous study,^11^ unilateral retinal function was recorded in Tg founder 53-1 using JET electrodes, a portable ERG unit (HMsERG model 1000; The Electrode Store, Buckley, WA), and mini-Ganzfeld dome positioned approximately 1 cm from the eye.

All pigs were euthanized with a solution containing pentobarbital sodium and phenytoin sodium (Beuthanasia-D, 1 mL/5 kg, i.v.; Intervet/Merck Animal Health, Millsboro, DE), and the eyes enucleated and immediately immersed in fixative for morphologic studies.

### Light Microscopy

Plastic sections of retinal tissue were prepared using a previously described technique.^13,14^ Briefly, 3 mm wide bands of retinal tissue were removed along vertical and horizontal axes from the optic nerve head to the peripheral margin of the retina. Retinal tissue was dehydrated in ascending ethanol concentrations, infiltrated and embedded in JB-4 Plus resin (Ted Pella, Redding, CA). Blocks of embedded retinal tissue were cut into 4 μm thick sections using a Leica EMUC6 Ultramicrotome (Leica Microsystems, Buffalo Grove, IL). Retinal sections were stained with 1% cresyl violet (Sigma-Aldrich Corp., St. Louis, MO) and examined at ×40 or ×100 using a NIKON EFD-3 Episcopic-Fluorescence microscope (Nikon, Inc., Melville, NY). Retinal photomicrographs were taken on a Moticam 2500 high-resolution camera (Motic, British Columbia, Canada) and digitally processed to adjust brightness and contrast using Adobe Photoshop (Adobe Systems, San Jose, CA). Retinal micrographs were taken from the area centralis, midperipheral, and peripheral retina in animals that exhibited a gradient of outer retinal degeneration, while retinal micrographs were taken from only the area centralis in animals that showed uniform degeneration across the entire retina.

Using previously established techniques^13,14^ to measure outer nuclear layer (ONL) thickness in wild type (WT) versus TgP23H. Mean overall ONL was compared across all ages using 1-way ANOVA and post hoc *t*-tests with a *P* value of ≤ 0.05 considered a significant difference. We used InStat 3 for Macintosh (GraphPad Software, Inc., La Jolla, CA) for statistical analyses of retinal morphometric data.

### Transmission Electron Microscopy (TEM)

Preparation of ultrathin sections of retinal tissue for examination by TEM has been described previously.^13,14^ Briefly, a 2 × 2 mm piece of retinal tissue was removed 5 mm above the superior margin of the optic disc. Retinal tissue was rinsed in buffer, postfixed in 2% osmium tetroxide and 1.5% potassium ferrocyanide in dH_2_O for 2 hours, dehydrated in a graded series of ethanols and embedded in Epon-Araldite (Electron Microscopy Sciences, Hatfield, PA). Ultrathin sections (90 nm) of embedded retinal tissue were cut from each block with a diamond knife (Micro Star Technologies, Inc., Huntsville, TX) using an ultramicrotome (Ultracut E 701704; Reichert-Jung, Buffalo, NY). Ultrathin sections were collected on copper grids, and counterstained with 4% methanolic uranyl acetate (Electron Microscopy Sciences). Photoreceptor structure was then examined with a transmission electron microscope (Model 300: Phillips, Eindhoven, The Netherlands).

### Immunohistochemistry

Sections 20 μm thick were cut on a cryostat and stored at −80°C until further processing. Frozen sections were removed from the freezer and then processed for immunohistochemical studies. Specificity of the primary and secondary antibodies has been described previously.^15^ Primary antisera included monoclonal anti-rho 1D4 ( Cat. #P21940, 1:1000; Thermo Fisher Scientific, Waltham, MA) to label rods, polyclonal anti-protein kinase C-α to label rod bipolar cells (Cat. # P 4334; Sigma-Aldrich Corp.), monoclonal anti-vimentin (clone # Clone V9) to label Müller cells (Cat. # V 6630, 1:32K; Sigma-Aldrich Corp.). Fluorophore-labeled secondary antibodies included Alexa Fluor 488 chicken anti-mouse IgG (H + L; Cat. # A21200, 1:10; Invitrogen, Carlsbad, CA), Alexa Fluor 647 chicken anti-mouse IgG (H + L; Cat. # A21463; Invitrogen), Alexa Fluor 488 donkey anti-rabbit IgG (H + L; Cat. # A21206, 1:10; Invitrogen, Carlsbad, CA, 1:10). After incubation in fluorophore-labeled secondary antibody serum, sections were rinsed in buffer and mounted in Vectashield mounting medium with 4′,6-diamidino-2-phenylindole (DAPI; Cat.# H-1200; Vectorlabs, Burlingame, CA). Immunolabeled retinal tissue was examined by confocal microscopy (Olympus FV1000). Control sections were not exposed to primary antibodies, but were processed simultaneously through all other labeling steps. These no-primary control sections were included in all labeling studies.

## Results

### Retinal Fundus Examination is Normal

Neither F1 Tg offspring nor Tg founder 53-1 exhibited the typical fundus changes that occur with disease progression in RP, such as intraretinal pigment migration (e.g., bone spicules), retinal arteriolar vascular attenuation, and pallor of the optic nerve head.

### Cone Driven Function Develops Normally in the Absence of Rod Driven Function

**Figure 1:**
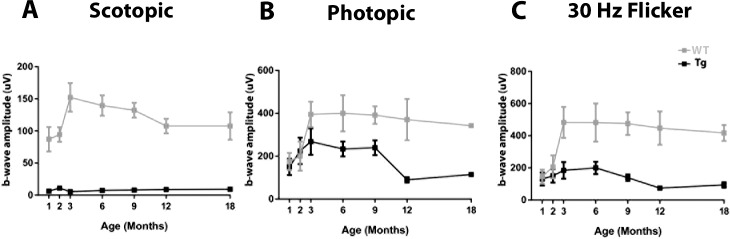
ff-ERG recordings for rod, cone, and 30Hz flicker. (**A**) The rod waveform never develops in the Tg swine (WT vs. Tg Scotopic ERG *P* values, 1 month 0.0055, 2 months 0.004, 3 months 0.0013, 6 months 0.0002, 9 months 0.0008, 12 months 0.0187, 18 months 0.0491) The cone and cone flicker waveforms are similar in WT and Tg up to 2 months, but then decline in Tg swine ([**B**] and [**C**], respectively). (WT vs, Tg Photopic ERG *P* values, 1 month 0.6642, 2 months 0.7998, 3 months 0.094, 6 months 0.0598, 9 months 0.0146, 12 months 0.0511, 18 months 0.0023; WT vs. Tg 30Hz Flicker *P* values, 1 month 0.8136, 2 months 0.5276, 3 months 0.0349, 6 months 0.0639, 9 months 0.0088, 12 months 0.0785, 18 months 0.0332).

To characterize retinal function in WT and Tg littermates, we used a standard ISCEV ffERG protocol.^16^ Figure 1 illustrates representative ERG responses to different flash intensities. Clearly evident are responses in WT miniswine to flashes at all intensities, whereas TgP23H miniswine lack an ERG response to a rod isolated stimulus (0.01 cd·s·m^−2^). A cone (light-adapted 3.0 cd·s·m^−2^) and a 30 Hz flicker stimulus of the ERG responses for WT and TgP23H miniswine are similar up to 2 months, then decline in TgP23H swine (*P* = 0.0119, *P* = 0.0005 respectively). These results suggested that rod driven retinal function never develops and cone driven function is affected beyond 2 months of age in this model of P23H retinopathy.

### Rod Photoreceptor Dropout Follows a Central-to-Peripheral Retinal Pattern

**Figure 2:**
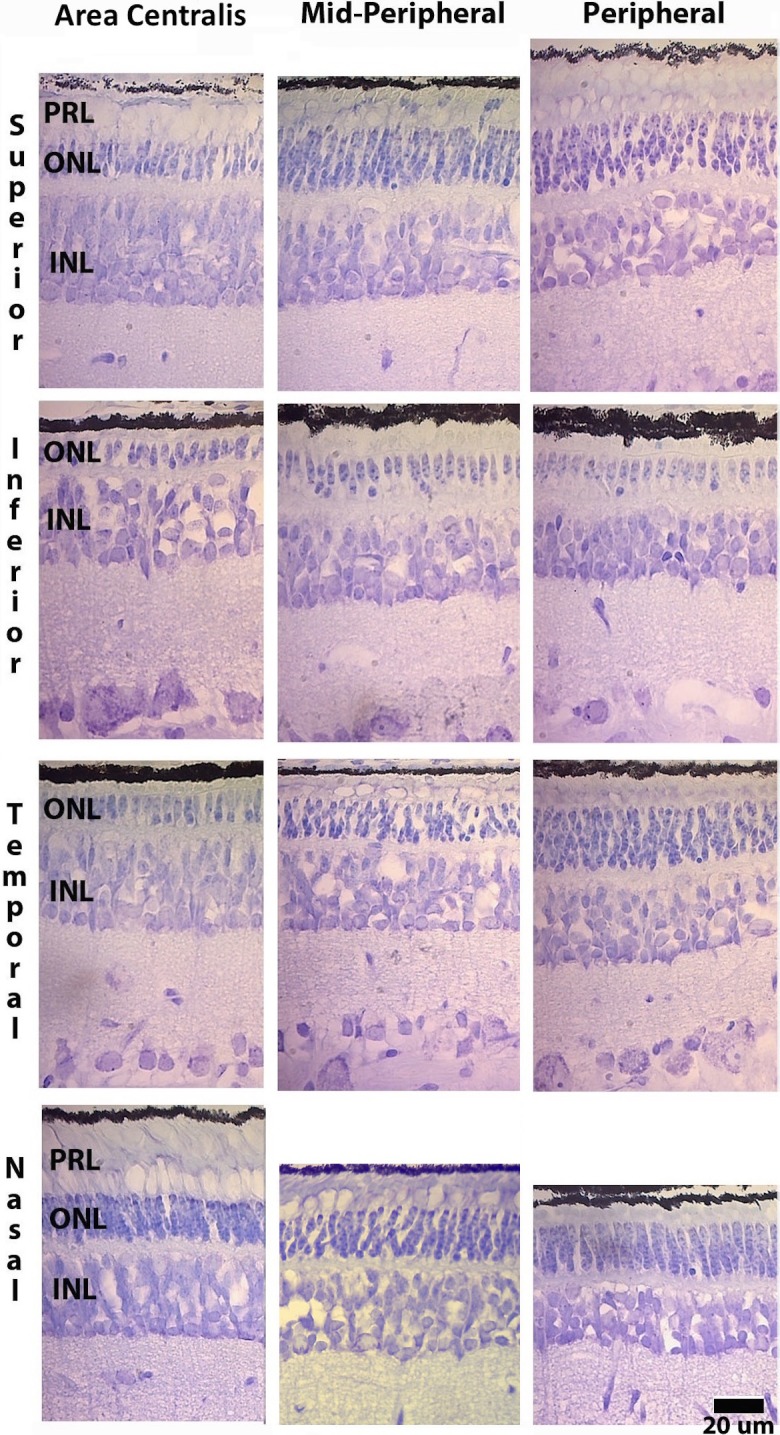
Retinal pattern of ONL thinning in Tg 1 month. A central-to-peripheral thinning of the ONL is apparent in the superior, temporal, and nasal retina, while the inferior retina is composed of only a monolayer of cone nuclei. The PRL appears disorganized, while the inner nuclear layer (INL) appears intact.

**Figure 3:**
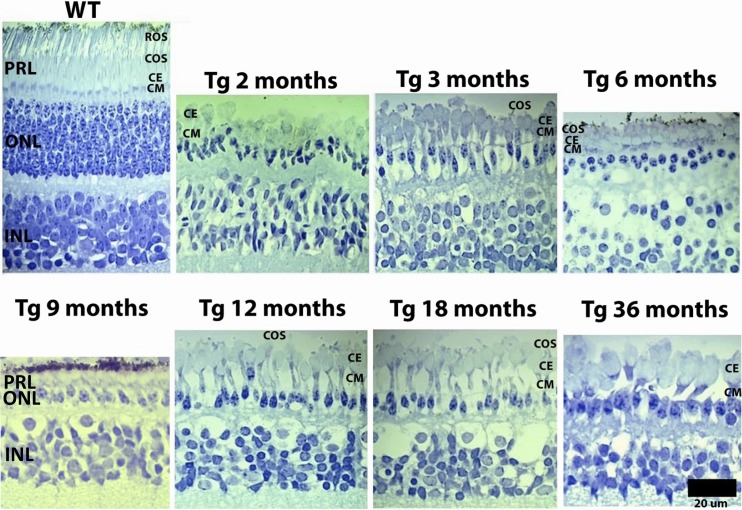
Light micrographs of outer retinal morphology. Wild type retinas show normal retinal morphology and appear similar across all WT littermates (not shown). Tg retinas show robust loss of photoreceptors from the ONL, with cone nuclei showing a discontinuous monolayer at 3 months, which appears continuous by 6 months of age. Cone outer segments (COS) were observed at 6 months, with infrequent COS present out to 12 months. Cone inner segments exhibit a tear drop-like shape in all Tg retinas, with the ce appearing enlarged and an elongated cm relative to WT retinas. The INL appears normal.

Loss of rod photoreceptor nuclei from the ONL was evident at 1 month in Tg retinas. Rod photoreceptor dropout followed a central-to-peripheral retinal pattern along the vertical and horizontal meridians, with the inferior retina appearing most severely affected such that eventually only a monolayer of cone nuclei remained in the ONL (Fig. 2). Remaining nuclei in the ONL were determined to be those of cone photoreceptors given their large size and abundance of euchromatin,^17^ as well as observable anatomic continuity of the cell membrane from the cone somata to the inner segment in the photoreceptor layer (PRL) as shown by electron microscopy. All rod photoreceptor nuclei were absent from the ONL by 3 months in Tg retinas (Fig. 3).

**Figure 4:**
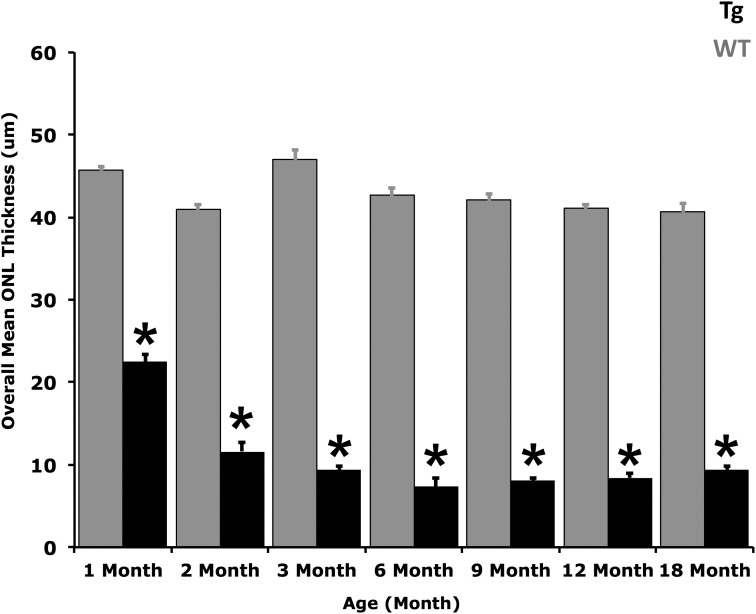
Histogram of mean overall ONL thickness. The histogram shows significant loss in the mean overall ONL thickness in TgP23H miniswine at all ages compared to WT littermates. *P value < 0.05.

Figure 4 shows a significant decrease (*P* value < 0.05) in the mean overall ONL thickness from 1 to 18 months in WT versus TgP23H littermates. No significant difference in ONL thickness was observed across all WT littermate retinas.

### Rod Photoreceptors Show Abnormal Localization of Rhodopsin

**Figure 5:**
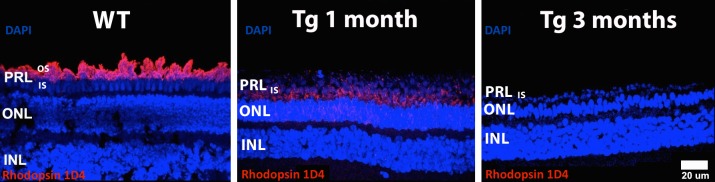
Immunolabeling with rhodopsin 1D4. 4′,6-Diamidino-2-phenylindole (DAPI) and rhodopsin 1D4 immunostaining (*red labeling*) in WT is confined to rod OS in the PRL and appear similar across all WT littermates (not shown), while 1 month Tg pigs show immunostaining in the rod IS and ONL, and no immunostaining at 3 months due to complete dropout of rod photoreceptors. IS, inner segment.

Immunolabeling of rod photoreceptors with anti-rhodopsin 1D4 antibody was restricted to the outer segment (OS) in all WT littermate retinas (Fig. 5, left panel, red labeling), while 1-month Tg retinas showed abnormal localization of rhodopsin to the inner segment (IS) and plasma membrane of the cell body in the ONL (Fig. 5, middle panel, red labeling). Rhodopsin was no longer detectable at 3 months in Tg retinas (Fig. 5, right panel).

### Cone Photoreceptors Remain in the ONL Although They Have Compromised Morphology

**Figure 6:**
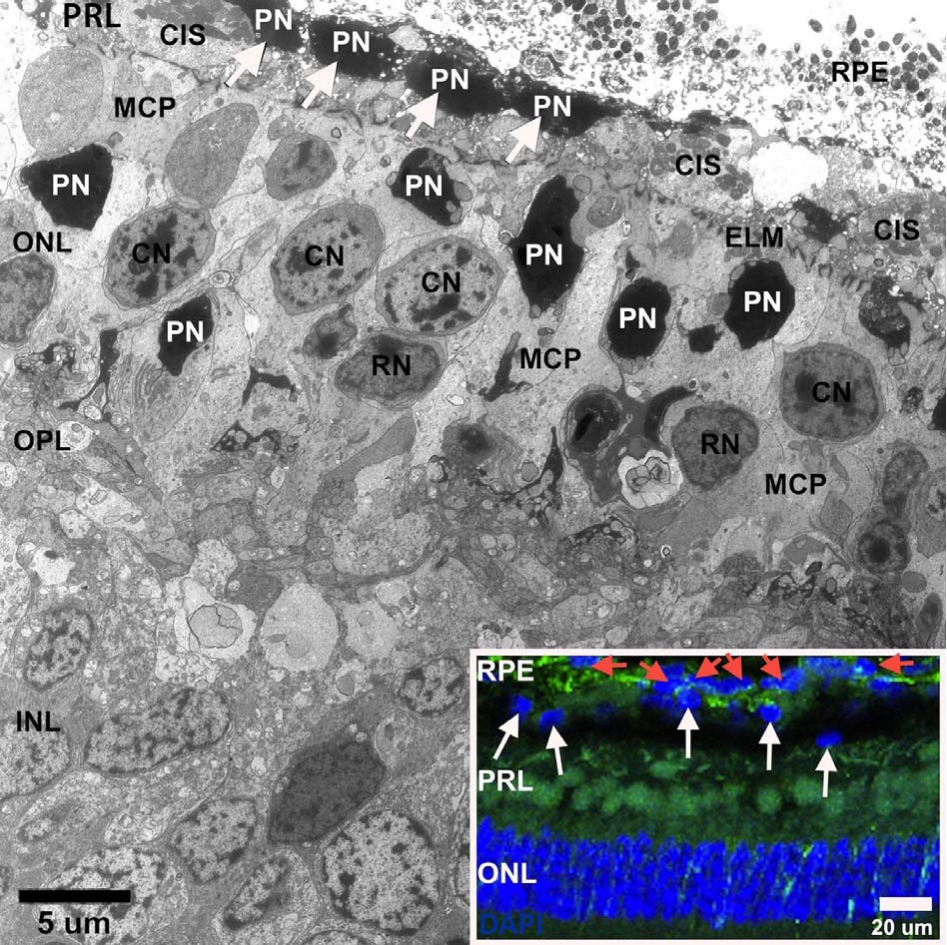
Ultrastructure of the outer retina in Tg 1 month. No rod or cone OS are seen in the PRL, CIS abut the RPE, and PN are seen along the RPE and within the ONL. The ELM is intact. The ONL is composed of rod nuclei (RN) and cone nuclei (CN) and appears disorganized with numerous hypertrophied MCP weaving between photoreceptor nuclei. *Inset*: DAPI stained photoreceptor nuclei (*white arrows*) are located in the PRL inward from nuclei of the RPE (*red arrows*). The *green* coloration is due to autofluorescence.

Cone OS were absent in most of the PRL between 1 and 2 months in Tg retinas, while cone inner segments (CIS) were present and abutted the RPE (Fig. 6). The ONL appeared disorganized, lacked regular stacking of photoreceptor nuclei into vertical columns, and showed numerous pyknotic nuclei (PN). Pyknotic nuclei-like structures also were observed in the PRL (Fig. 6, white arrows) situated between the external limiting membrane (ELM) and underlying RPE, and may represent degenerating nuclei of photoreceptors. Staining with DAPI, a fluorescent stain that binds strongly to DNA, further suggests these PN-like structures in the PRL may be degenerating photoreceptor nuclei (Fig. 6, inset, white arrows).

**Figure 7:**
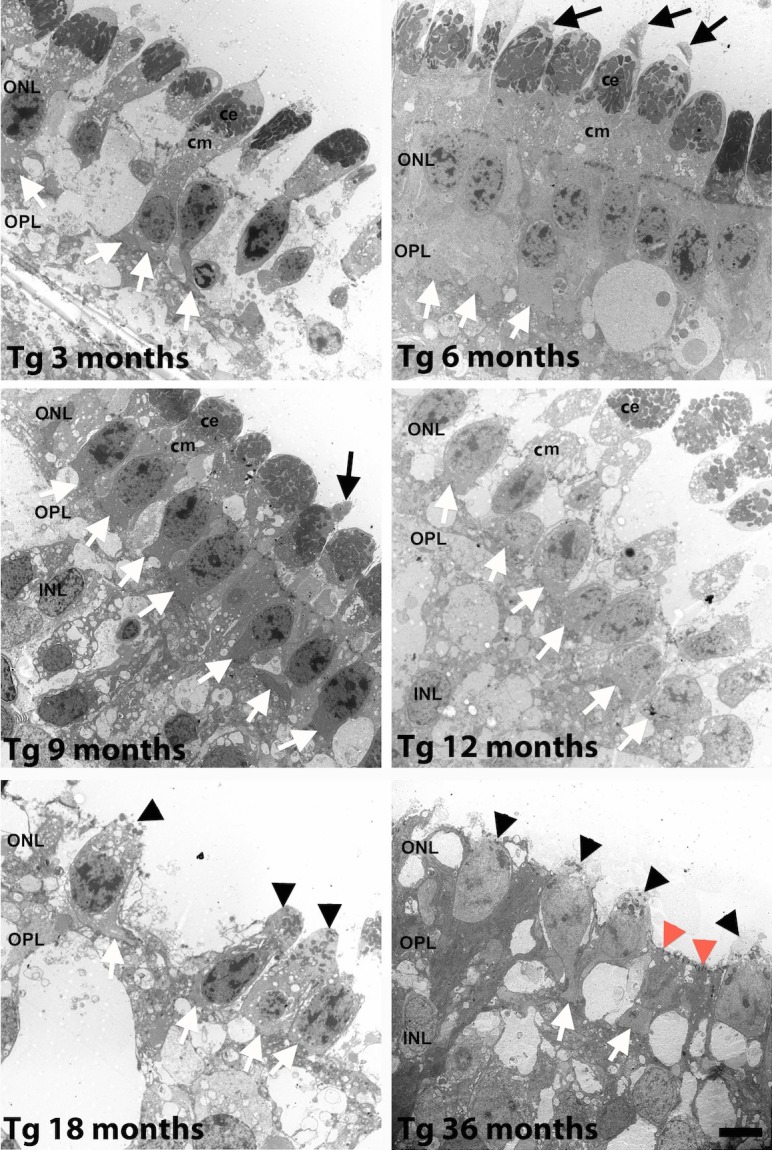
Transmission electron micrographs of cone photoreceptors. At 6 months cone OS (*black arrows*) were observed, the ce, cm, and cone pedicle (*white arrows*) were present. At 12 months, cone pedicles (*white arrows*) closer to the cell body. At 18 and 36 months the ce (*black arrowheads*) and cm are retracted, ce contain few mitochondria, there are few cone pedicles (*white arrows*), and zonula adherens junctions appear discontinuous along the ELM. *Scale bar*: 5 um.

In 3-month Tg retinas (Fig. 7), cone photoreceptors lacked outer segments, while the axon and pedicle (Fig. 7, white arrows) appeared close to the cell body. The cone myoid (cm) and cone ellipsoid (ce) of the inner segment appeared normal.

In 6-month Tg retinas (Fig. 7), cone photoreceptors exhibited normal inner and outer segments (black arrows), pedicles (white arrows) appeared close to the cone somata, and cone somata formed a continuous monolayer in the ONL.

In 9-month Tg retinas (Fig. 7) most cone photoreceptors lacked an outer segment (black arrow) and the ce and cm exhibited normal morphology.

In 12- and 18-month Tg retinas (Fig. 7), the ELM is absent and the cone inner segment has retracted towards the cell body and shows fewer mitochondria in the cone ellipsoid (black arrowheads).

In 36-month Tg retinas (Fig. 7), cone inner segments (black arrows) were largely obliterated and those remaining showed few mitochondria that were encroaching upon the cell body. Few cone photoreceptors showed axons and pedicles (white arrows). The ELM was largely discontinuous, with few zonula adherens junctions (red arrows) remaining intact.

### Dendritic Retraction of Rod Bipolar Cells

**Figure 8:**
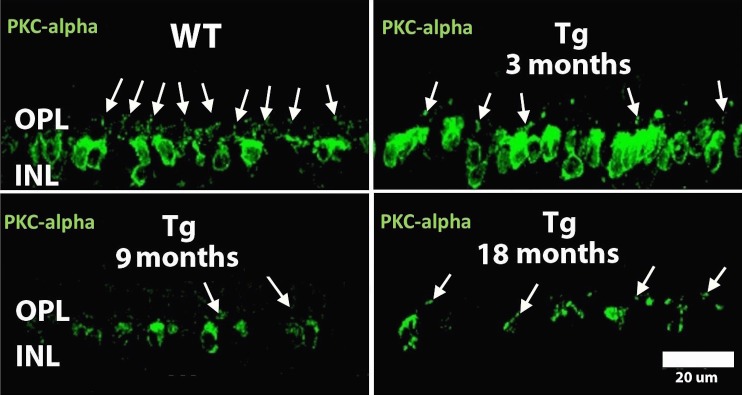
Immunolabeling with PKC-α. Wild type show protein kinase-a (PKC-a) immunostaining (*green labeling*) of rod bipolar cell dendrites (*white arrows*) in the OPL and cell body in the INL and appear similar across at WT littermates (not shown), while Tg retinas show a progressive decrease in immunoreactivity in the OPL (*white arrows*) and INL.

Anti-PKC-α immunolabeling (Fig. 8, green labeling) of rod bipolar cell bodies and dendrites appeared similar across all WT littermate retinas and Tg at 1 month (not shown). Retraction of rod bipolar cell dendrites from the outer plexiform layer (OPL) was evident by 3 months (Fig. 8, white arrows) and was more apparent at 9 months and beyond in Tg retinas.

### Gliosis of the Outer Retina

**Figure 9:**
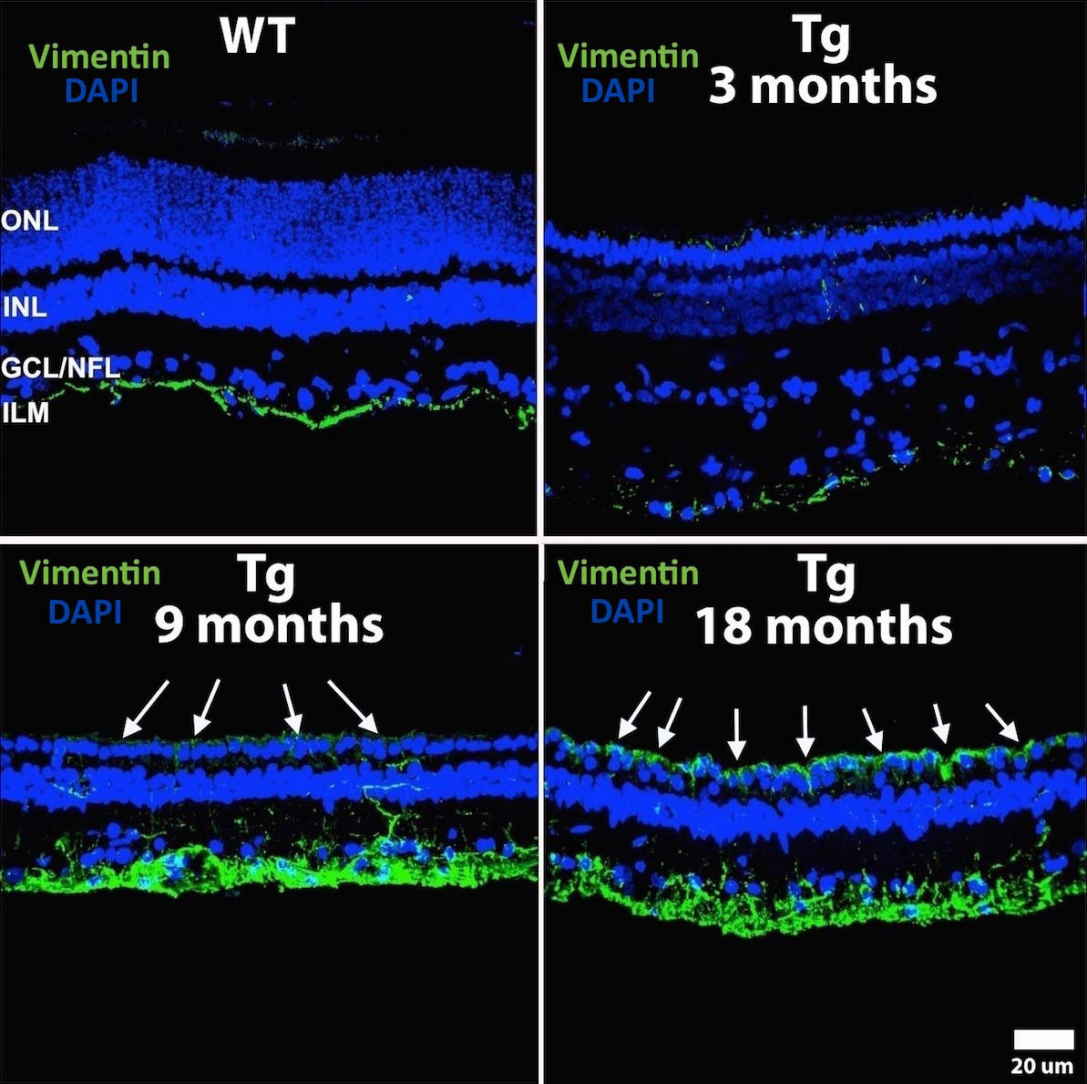
Immunolabeling with vimentin. 4′,6-Diamidino-2-phenylindole (DAPI) and vimentin immunolabeling (*green labeling*) of Müller cells in appeared similar across all WT littermates (not shown) and similar to Tg retinas up to 6 months. Tg retinas at 9 months show increased immunolabeling of Müller cell footplates and their inner stalks, as well as mild immunolabeling in the ONL, and formation of a glial seal (*white arrows*) at 18 months. GCL/NFL, ganglion cell layer/nerve fiber layer; ILM, internal limiting membrane.

Hypertrophied Müller cell processes (MCP) were seen throughout the retina surrounding cone cell bodies in the ONL in 1 month Tg retinas (Fig. 6); however, only after structural integrity of the ELM was compromised did we observed outer retinal gliosis (Fig. 9, bottom panels, white arrows), as evidenced by immunolabeling with anti-vimentin antibody (green labeling) in 18 month-old Tg retinas. Immunolabeling with anti-vimentin appeared similar across all WT littermates (Fig. 6).

### Cone Photoreceptors Remain in the Area Centralis at Six Years of Age in TgP23H Founder 53-1

**Figure 10:**
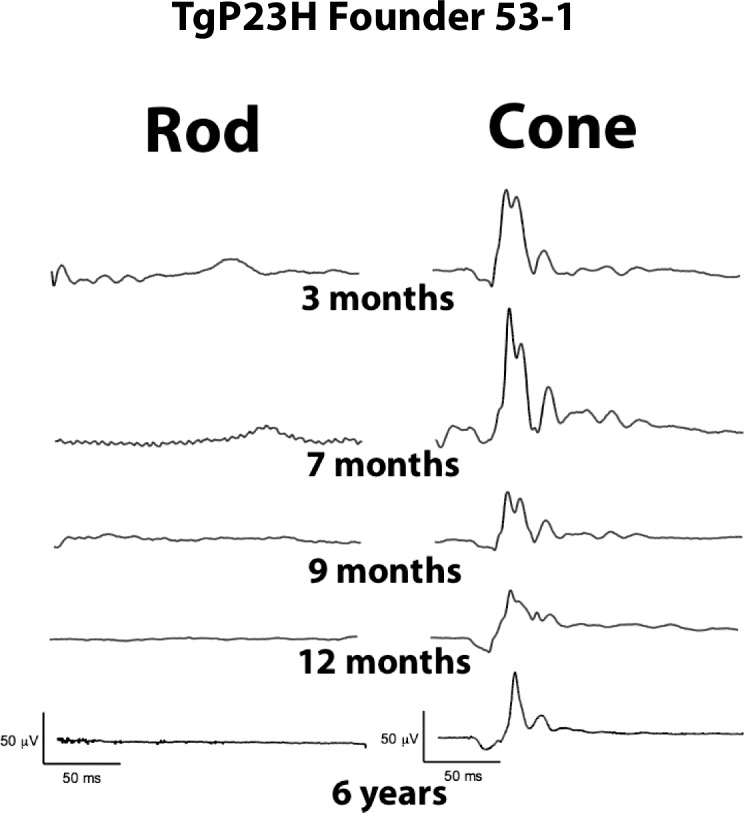
ff-ERG rod and cone responses in TgP23H founder 53-1. There is no rod waveform in this Tg founder at any of the tested time points. In contrast there is a cone waveform that is present, albeit diminished, until 6 years of age.

The full field ERG responses from the 53-1 founder (Fig. 10) show an absence of a rod response at any of the tested time points, between 3 months and 6 years of age. In contrast there is a cone response at all ages with amplitudes that decrease in the first months but then remain unchanged up to the last time point when the animal was euthanized.

**Figure 11:**
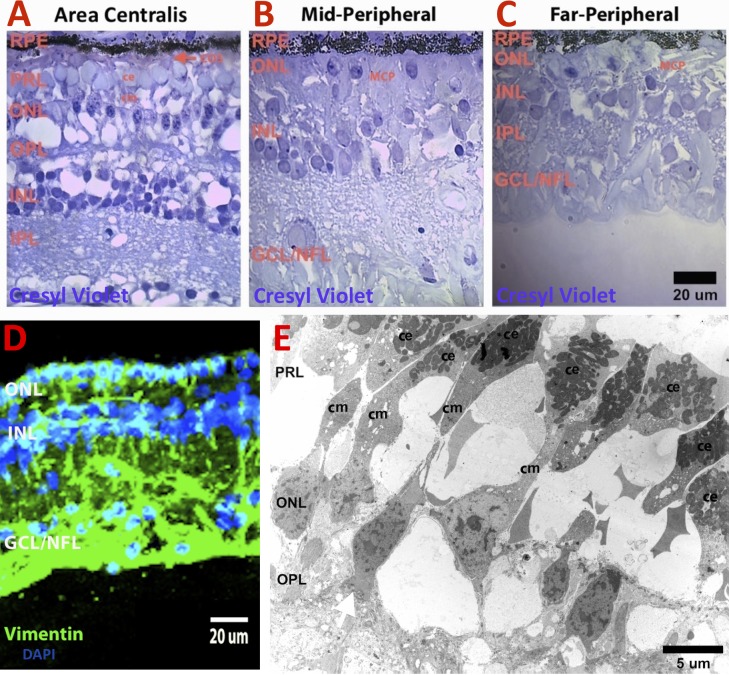
Retinal morphology of TgP23H founder (53-1) miniswine at 6 years of age. (**A**–**C**) Light microscopy. (**D**) Confocal microscopy. (**E**) Transmission electron microscopy. All the layers of the outer and inner retina were stained with cresyl violet 1% could be identified in the area centralis (**A**). A single COS (*red arrow*) was found. The PRL and OPL could not be identified in the mid-peripheral (**B**) and peripheral retina (**C**) due to severe gliosis of the outer and inner retina as evidenced by immunolabeling with anti-vimentin antibody and confocal microscopy (**D**). At the electron microscopic level (**E**), surviving cone photoreceptors in the area centralis showed intact inner segments with abundant mitochondria in the CE and an elongated cm, as well as axons with an occasional pedicle (*white arrow*). IPL, inner plexiform layer, ONL, outer nuclear layer.

By light microscopy (Fig. 11A), all the layers of the outer and inner retina could be identified in the area centralis. A few cone outer segments (Fig. 11A, red arrow) were seen by light microscopy in the area centralis. However, the PRL and OPL could not be identified in the mid-peripheral (Fig. 11B) and peripheral (Fig. 11C) retina due to severe gliosis of the outer and inner retina as shown by immunolabeling with anti-vimentin antibody and confocal microscopy (Fig. 11D).

At the electron microscopic level (Fig. 11E), surviving cone photoreceptors in the area centralis showed intact inner segments with abundant mitochondria in the cone ellipsoid and an elongated cone myoid, as well as axons with an occasional pedicle (Fig. 11C, white arrow).

## Discussion

In the present study, we showed that F1 TgP23H miniature swine exhibited a similar progressive retinopathy as observed in the TgP23H miniswine founder 53-1,^11^ and that cone photoreceptors with compromised morphology remained after 6 years in the area centralis of the founder and produced a barely detectable photopic visual signal in the severely degenerated retina.

Rapid onset and robust rod degeneration in F1 TgP23H miniswine does not affect development of cone driven function until after 2 months of age, even though many cone photoreceptors have lost their outer segments at 1 month. Although cone morphology is altered they remain partially functional. The partial function is enough to generate a photopic response similar to that in developing WT. The subsequent plateau of cone function development (Fig. 1, 3–9 months) is followed by a progressive decline (Fig. 1, 9–12 months) in F1 TgP23H miniswine and mirrors changes in cone morphology (Fig. 7) that include deformation of cone outer segments and their subsequent loss, as well as reorganization of cone nuclei into a monolayer, and subsequent disorganization and entombment by Müller cells. In the *rd1* mouse similar morphologic changes occur to cone photoreceptors, such as loss of cone outer segments and pedicles shortly after initiation of rod photoreceptor degeneration.^18^

In F1 TgP23H miniswine, numerous surviving cone photoreceptors showed restored CIS and COS morphology that coincided with the plateau period of cone function. This suggests cone photoreceptors in F1 TgP23H miniswine do not undergo rapid robust remodeling and that their neural circuitry remains intact longer than expected. In support of this, we found cone photoreceptors with intact inner segments and pedicles and a measurable photopic visual signal in our most severely affected TgP23H founder 53-1 at 6 years of age. Those surviving cone photoreceptors with intact inner segments and pedicles were exclusive to the area centralis, which is similar to retinal pattern of cone photoreceptor degeneration in end-stage human RP.^19^ Moreover, the morphologic changes to cone photoreceptors in the area centralis are similar to those observed in the foveal slope in patients with end-stage autosomal dominant RP.^20^ The presence of a cone-rich area of retinal specialization in this model, which is absent in rodents, may be important for recapitulating the geographic progression of cone photoreceptor degeneration that occurs in humans with RP.

The central-to-peripheral pattern of rod photoreceptor degeneration and abnormal localization of rhodopsin is consistent with our previous study in TgP23H hybrid miniswine embryos and newborn piglets.^14^ In the present study, however, the inferior retina showed a uniform pattern of retinal degeneration with only a monolayer of cone nuclei remaining in the ONL. The severity of retinal damage in the inferior retina may be related to environmental stress factors, such as phototoxicity from overhead lighting, which may affect the spatial pattern and tempo of retinal degeneration.^21,22^ Similar to TgP23H miniswine, the inferior retina in some patients with P23H retinopathy exhibit severe degeneration, which may be a result of phototoxicity from overhead light sources.^23^

The TgP23H miniswine model of RP recapitulates many features of human Pro23His retinopathy and will be very useful for developing therapeutic intervention strategies that translate to humans with RP. The use of the TgP23H swine model of RP will vary based upon the biological target and therapeutic approach. Therapeutic interventions, such as gene therapy and cell transplantation, that target rod photoreceptors and scotopic vision will need to be implemented before or shortly after birth due to the early onset and rapid degeneration of rod photoreceptors in this model, while therapeutic interventions that target cone photoreceptors and photopic vision have a delayed window of opportunity due to protracted degeneration of cone photoreceptors. Furthermore, the absence of retinal gliosis in the outer retina until very late in the disease provides a broad window of opportunity for delivery of trophic factors and gene therapy to cone photoreceptors, as well as for cell transplantation and integration, which would otherwise be impeded by the glial seal.

In recent experiments^13,14,24^ performed using the porcine model of Pro23His retinopathy we used *hybrid* TgP23H recipients, using semen from TgP23H miniswine^11^ to inseminate domestic sows. These litters were much larger than those obtained using miniswine and developed a similar pattern of retinal degeneration although some important differences in tempo were noted. Briefly, both *hybrid* TgP23H and F1 TgP23H miniswine develop a normal photopic response despite the absence of functional rod photoreceptors and their rapid loss from the ONL.^13,14^ However, degenerative changes to cone photoreceptor morphology, such as loss of outer segments and the absence of ribbon synapses in pedicles occurred earlier in the F1 TgP23H miniswine versus *hybrid* TgP23H swine. The variability in cone photoreceptor degeneration observed in the TgP23H miniswine represents biologic variability, since offspring are derived from insemination of wild type sows with P23H Tg semen, and these sows have a heterologous genetic background.^11^ Variation in gene penetrance is a characteristic feature of inherited diseases and is regularly observed in families in which the RP gene mutation is identical, but the disease has variable expression in offspring because their genetic background is different despite having the same parents. Despite these changes in cone morphology, both models show that partial photopic function remains. Further studies examining the neural circuitry downstream to cone photoreceptors in these models is warranted.

In conclusion, F1 TgP23H miniswine exhibited an RP phenotype similar to their founder and humans with Pro23His retinopathy. The TgP23H miniswine model will be a useful large eye model for developing therapeutic strategies that preserve/delay photopic vision loss in humans with RP.
